# Effects of dietary supplementation with a carvacrol–cinnamaldehyde–thymol blend on growth performance and intestinal health of nursery pigs

**DOI:** 10.1186/s40813-023-00317-x

**Published:** 2023-05-23

**Authors:** Bi-Chen Zhao, Tian-Hao Wang, Jian Chen, Bai-Hao Qiu, Ya-Ru Xu, Qing Zhang, Jian-Jie Li, Chun-Jiang Wang, Qiu-Feng Nie, Jin-Long Li

**Affiliations:** 1 College of Veterinary Medicine, Northeast Agricultural University, Harbin, 150030 P. R. China; 2 Heilongjiang Key Laboratory for Laboratory Animals and Comparative Medicine, Northeast Agricultural University, Harbin, 150030 P. R. China; 3 Key Laboratory of the Provincial Education Department of Heilongjiang for Common Animal Disease Prevention and Treatment, Northeast Agricultural University, Harbin, 150030 P. R. China; 4 Weiyuan Animal Pharmaceutical Co., Ltd., Shijiangzhuang, 052165 P. R. China

**Keywords:** Essential oil, Growth promoter, Intestinal health, Intestinal morphology, Nursery pig

## Abstract

**Background:**

Stress, herd transfer, and food changes experienced by nursery and fattening pigs can lead to reduced performance, reduced digestion and absorption, and impaired intestinal health. Given the role of essential oils in relieving stress and improving animal welfare, we hypothesized that essential oils may improve pig performance via promoting gut health and gut homeostasis laid by EOs supplementation during nursery continuously impacts performance in fattening pigs.

**Results:**

A total of 100 piglets (Landrace × Large White; weighted 8.08 ± 0.34 kg, weaned at d 28) were randomly selected and divided into 2 treatments: (1) basal diet (**Con**); (2) basal diet supplement with 0.1% complex essential oils (**CEO**). The experiment period was 42 days. Then weaned piglets’ growth performance and indications of intestinal health were assessed. Compared to the Con group, dietary supplemented CEO enhanced BW at 14 d (*P* < 0.05), and increased ADG during 1 ~ 14 d and 1 ~ 42 d (*P* < 0.05). Furthermore, CEO group had lower FCR during 1 ~ 42 d (*P* < 0.05). The CEO group also showed higher VH and VH:CD in duodenum and ileum (*P* < 0.05). Additionally, dietary CEO supplementation improved gut barrier function, as manifested by increased the mRNA expression of tight-junction protein and decreased serum DAO, ET and D-LA levels (*P* < 0.05). Finally, CEO supplementation alleviated gut inflammation, increased the activity of digestive enzymes. Importantly, piglets supplemented with CEOs during nursery also had better performance during fattening, suggesting that the establishment of intestinal health will also continuously affect subsequent digestion and absorption capacity. In short, dietary supplemented CEO improved performance and gut health via modulating increased intestine absorptive area, barrier integrity, digestive enzyme activity, and attenuating intestine inflammation. Meanwhile, essential oil supplementation during the nursery period also had a favorable effect on the performance of growing pigs.

**Conclusions:**

Therefore, the strategy of adding CEO to pig diets as a growth promoter and enhancing intestinal health is feasible.

**Supplementary Information:**

The online version contains supplementary material available at 10.1186/s40813-023-00317-x.

## Background

The nursery period is a critical period for promoting piglet health and achieving optimal performance throughout life. Early weaning experienced by piglets at the beginning of the nursery period is a common practice to facilitate feeding management and reduce the cost in intensive pig production. But premature weaning reduces health and growth performance, making pigs susceptible to disease [1]. Weaning is a sudden, stressful and complex event characterized by changes in dietary, social and environmental living conditions, which profoundly affect piglet health throughout the nursery period, resulting in decreased performance and animal welfare [2]. The transition from easily digestible liquid milk to less digestible solid feed has a negative impact on the behavior of piglets and their immature fragile gastrointestinal tract as well [3]. The performance of nursery pigs was improved by the application of subclinical dosages of antibiotics [4]. Silva Júnior et al. reported that antibiotics improve intestinal health and maximize the genetic potential of animals, lowering diarrhea induced by subclinical intestinal diseases [5]. However, the problem of bacterial resistance and food-borne antibiotic residues caused by the abuse of antibiotics has aroused much concern, and many countries have banned antibiotics as growth promoters [6]. At present, the issues of intestinal sub-health and insufficient production performance of nursery pigs have not been properly resolved after the ban on antibiotics [7]. Therefore, exploring growth promoters that can maintain piglet performance at the nursery period while protecting public health has become an urgent priority.

Essential oils (EOs) are bioactive compounds extracted from plants that are widely used as sensory additives in human and animal feeds [8]. Recent studies suggested that the antioxidant properties of essential oils can safeguard the quality of food and inhibit the release of spoilage odors from the diet, thereby improving the palatability of the diet [9, 10]. Cinnamaldehyde, carvacrol, and thymol are the three most widely used and well-studied EOs, not only commonly used as feed flavoring agents, but have also been shown benefits such as promoted faster gut maturation, better nutrient digestibility, and improved animal performance [11]. Essential oils are mainly used in multiple combinations to achieve the best practical effect through compatibility [12]. Yang et al. reported that adding plant extract combinations (including cinnamaldehyde, thymol) to diets of early weaned pigs significantly increased BW and ADG [13]. Dietary supplementation of carvacrol and thymol also alleviated inflammation and increased the length of intestinal villi in weaned pigs [14]. These studies demonstrated a potential role for EO in promoting performance and intestine health. Establishing good intestinal stability during nursing period is conducive to ensuring the growth and development of fattening pigs and achieve excellent performance. In addition, farmers are generally reluctant to continue to supplement essential oil feed additives during the fattening period because of feeding costs. The hypothesis to be tested in this study is whether a mixture of carvacrol, cinnamaldehyde, and thymol of EOs improves performance and gut health in nursery pigs, and whether gut homeostasis laid by EOs supplementation during nursery continuously impacts performance in fattening pigs.

## Results

### Piglet performance and hematology blood biochemistry

The performance data in biweekly throughout the trial period were taken to assess changes under different feeding regimes. The growth performance parameters of the piglets are summarized in Table 1. Piglets fed with CEO showed numerically increased BW at 14 d (*P* < 0.05) in comparison with that on the basal diet. Compared with Con group, CEO group had lower FCR during 29 ~ 42 d (*P* = 0.067) and 1 ~ 42 d (*P* < 0.05). Besides, piglets fed CEO diet had higher ADG during 1 ~ 14 d (*P* < 0.05) and 1 ~ 42 d (*P* < 0.01), when compared to piglets fed Con diet. At every phase, there was no appreciable difference between the dietary treatments for ADFI, diarrhea rate, and diarrhea index. Organ pictures and organ coefficients for liver, lung and spleen are shown in Supplementary Fig. S2. The organ coefficients of liver, lung and spleen did not substantially differ between the two groups. In addition, compared with the control group, the piglets’ body weight (*P* < 0.01) and gut length (*P* < 0.05) increased significantly after CEO supplementation, while the ratio of gut length to body weight was not significantly different, suggesting a positive correlation between gut length and body weight. The hematology blood biochemistry of the piglets is shown in Table 2. Dietary CEO supplementation did not significantly influence the blood biochemistry parameters of the nursery piglets.

**Table 1:** Growth performance of nursery piglets as affected by dietary CEO supplementation^1^

Item	Con^2^	CEO	SEM	*P-value*
1 d BW, kg	8.20	8.08	0.06	0.372
14 d BW, kg	11.93	12.79	0.29	0.038
28 d BW, kg	19.43	19.91	0.24	0.117
42 d BW, kg	29.16	30.81	0.82	0.077
1 ~ 14 d				
ADG, g/d	264.29	340.71	38.21	0.084
ADFI, g/d	338.16	399.54	30.69	0.060
FCR	1.286	1.19	0.05	0.527
Diarrhea rate, %	20.77	14.16	3.30	0.072
Diarrhea index	1.07	0.88	0.10	0.080
15 ~ 28 d				
ADG, g/d	451.62	488.543	18.46	0.038
ADFI, g/d	664.49	703.38	19.44	0.213
FCR	1.48	1.44	0.02	0.745
Diarrhea rate, %	14.89	11.58	1.65	0.118
Diarrhea index	0.63	0.56	0.036	0.429
29 ~ 42 d				
ADG, g/d	748.86	774.07	12.61	0.572
ADFI, g/d	1218.13	1331.27	56.57	0.447
FCR	1.86	1.57	0.15	0.067
Diarrhea rate, %	11.53	8.84	1.35	0.069
Diarrhea index	0.56	0.39	0.08	0.067
1 ~ 42 d				
ADG, g/d	488.25	556.67	34.21	0.002
ADFI, g/d	740.26	811.39	35.57	0.147
FCR	1.68	1.43	0.13	0.020
Diarrhea rate, %	16.96	12.00	2.48	0.080
Diarrhea index	0.75	0.63	0.06	0.122

^1^Body weight (BW), average daily gain (ADG), average daily feed intake (ADFI), feed conversion ratio (FCR)

^2^Control (Con): a based diet. CEO: Con + compound essential oil (contain 5% cinnamaldehyde, 5% carvacrol and 0.7% thymol). N = 100 total, n = 50 per supplementation

**Table 2:** Hematology blood biochemistry of nursery piglets as affected by dietary CEO supplementation^1^

Item	Con^2^	CEO	SEM	*P-value*	Reference ranges
WBC, 10^9^/L	27.30	27.78	0.24	0.841	11.6 ~ 39.7
RBC, 10^12^/L	7.14	6.90	0.12	0.627	1.4 ~ 8.9
HGB, g/L	114.80	111.40	1.70	0.608	25.0 ~ 152.0
HCT, %	38.72	38.78	0.03	0.984	8.2 ~ 49.5
MCV, fL	54.40	56.24	0.92	0.236	48.1 ~ 68.7
MCH, pg	16.04	16.16	0.06	0.853	15.0 ~ 19.5
PLT, 10^9^/L	446.80	502.80	28.00	0.268	27.0 ~ 529.0
MPV, fL	7.08	6.92	0.08	0.628	5.6 ~ 11.3
PCT, %	0.33	0.33	0.01	0.844	0.0 ~ 0.5

^1^ White blood cell (WB), red blood (RBC), hemoglobin (HGB), hematocrit (HCT), mean corpuscular volume (MCV), mean corpuscular hemoglobin (MCH), platelet (PLT), mean platelet volume (MPV), thrombocytocrit (PCT)

^2^Control (Con): a based diet. CEO: Con + compound essential oil (contain 5% cinnamaldehyde, 5% carvacrol and 0.7% thymol). N = 100 total, n = 50 per supplementation

### Piglet pH value of each intestinal segment

Dietary CEO supplementation did not significantly affect the piglets’ pH value of each intestinal segment (Table 3).

**Table 3:** pH value of intestinal segment of piglets as affected by dietary CEO supplementation

Item	Con	CEO	SEM	*P-value*
pH				
Duodenum	6.32	5.87	0.22	0.125
Jejunum	6.39	6.17	0.11	0.319
Ileum	7.01	6.94	0.04	0.675
Cecum	6.34	6.18	0.08	0.543
Colon	6.24	6.15	0.04	0.655

### Gastrointestinal histological analysis of the nursery pigs

**Fig. 1:**
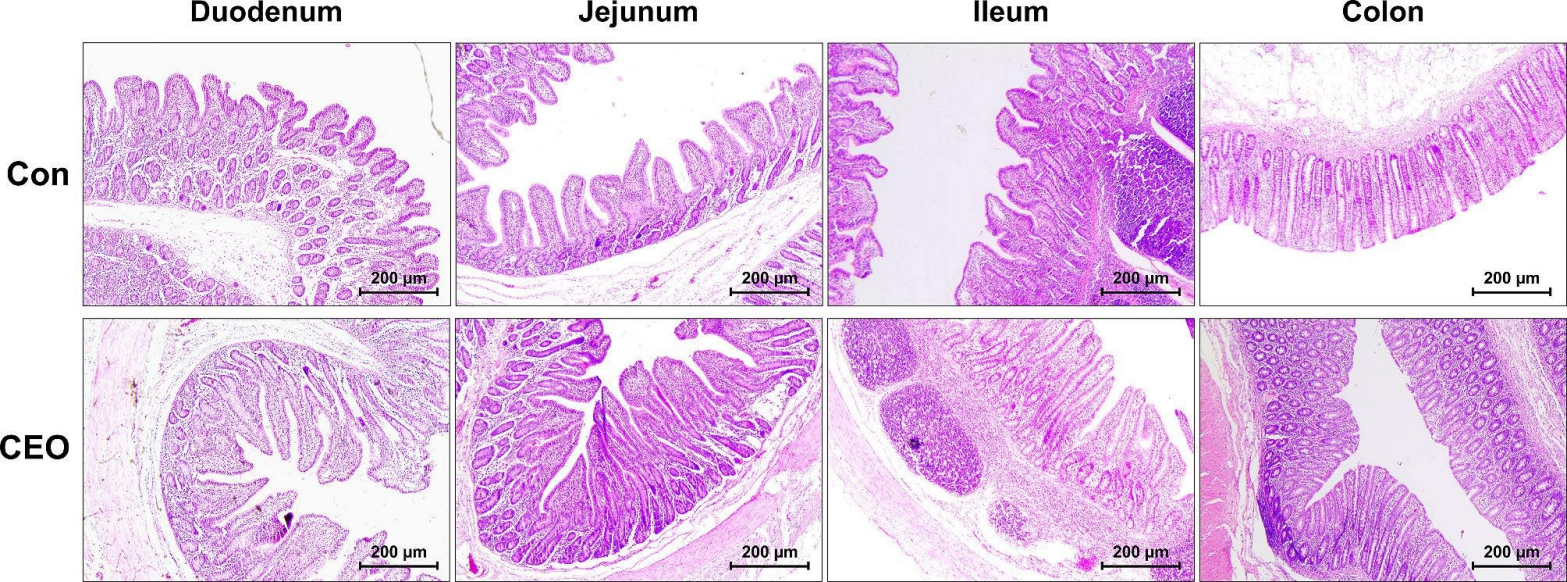
Effect of dietary CEO supplementation on intestinal morphology in nursery pigs by H&E staining

**Fig. 2:**
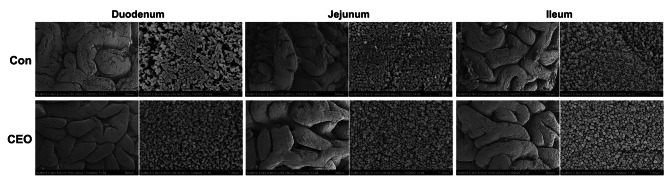
Effect of dietary CEO supplementation on intestinal microscopic morphology shown in nursery pigs by SEM

The intestinal morphometric indices of the nursery pigs are compiled in Table 4, and Fig. 1 displays the morphological properties of intestinal tissues taken from the nursery pigs in the various treatment groups. The VH and VH:CD of duodenum and ileum were significantly increased in the CEO group compared to piglets on basal diet with no added CEO (*P* < 0.05), indicating a significant increase in the absorption area of the intestine. In addition, microvilli were more densely and regularly arranged in the duodenum, jejunum, and ileum in the CEO group (Fig. 2).

**Table 4:** Intestinal morphometric indices of piglets as affected by dietary CEO supplementation^1^

Item	Con	CEO	SEM	*P-value*
Duodenum				
VH, µm	212.16	465.57	126.70	< 0.001
CD, µm	474.38	297.53	88.42	0.005
VH:CD	0.45	1.75	0.65	< 0.001
Jejunum				
VH, µm	289.18	397.67	54.24	< 0.001
CD, µm	186.27	303.69	58.71	0.001
VH:CD	1.48	1.35	0.07	0.364
Ileum				
VH, µm	353.25	449.79	48.27	0.011
CD, µm	344.27	227.72	58.27	0.017
VH:CD	1.08	2.07	0.50	0.002
Colon				
VH, µm	465.16	552.06	43.45	0.004

^1^Villus height (VH), crypt depth (CD), villus height to crypt depth ratio (VH:CD)

### Relative mRNA expression of genes involved in intestinal barrier function

The relative mRNA expression levels of ZO-1, caudin-1, and caudin-5 were superior in the duodenum and ileum of nursery pigs supplemented with CEO than in the Con group, respectively (*P* < 0.05, Table 5). In contrast, CEO-supplemented piglets showed an up-regulation of relative mRNA expression levels of occludin in the duodenum and jejunum, as well as E-cadherin in the jejunum and ileum (*P* < 0.05).

**Table 5:** The relative mRNA expression of intestinal epithelial integrity-related genes as affected by dietary CEO supplementation ^1^

Item	Con	CEO	SEM	*P-*value
Duodenum				
ZO-1	1.00	1.98	0.49	0.025
Claudin-1	1.00	1.93	0.47	0.035
Claudin-5	1.00	2.04	0.52	0.014
Occludin	1.00	2.07	0.53	0.012
E-cadherin	1.00	1.30	0.15	0.904
Jejunum				
ZO-1	1.00	1.10	0.05	0.911
Claudin-1	1.00	1.13	0.07	0.797
Claudin-5	1.00	1.28	0.14	0.109
Occludin	1.00	1.93	0.46	< 0.001
E-cadherin	1.00	2.30	0.65	< 0.001
Ileum				
ZO-1	1.00	2.57	0.79	< 0.001
Claudin-1	1.00	3.22	1.11	< 0.001
Claudin-5	1.00	2.00	0.50	< 0.001
Occludin	1.00	1.33	0.17	0.366
E-cadherin	1.00	1.88	0.44	< 0.001

^1^ The data were analyzed following the 2^−ΔΔCt^ method, and then the target gene transcript levels were normalized to β-actin

### Indicator of intestinal permeability in serum

Supplementation of CEO in the feed dramatically reduced the serum levels of DAO, ET and D-LA (*P* < 0.05, Table 6), which are key indicators for the evaluation of intestinal epithelial barrier function.

**Table 6:** The serum content of DAO, ET and D-LA as affected by dietary CEO supplementation^1^

Item	Con	CEO	SEM	*P-*value
DAO, pg/mL	201.53	124.28	38.63	0.004
ET, ng/L	253.67	180.46	36.61	< 0.001
D-LA, µg/L	715.70	644.05	35.83	0.044

^1^ Diamine oxidase (DAO); endothelin (ET); D-lactic acid (D-LA)

### Indicator of intestinal local inflammation in colonic mucosal

The improvement of intestinal barrier function integrity can reduce the effect of pathogenic bacteria on local intestinal inflammation, and the results showed that the contents of IL-1β, TNFα, and IL-6 in the colonic mucosal of nursery pigs in the CEO group were significantly decreased (Table 7, *P* < 0.05).

**Table 7:** The colonic mucosa content of IL-1β, TNFα and IL-6 as affected by dietary CEO supplementation

Item	Con	CEO	SEM	*P-*value
IL-1β, ng/mg protein	0.0181	0.0148	0.0016	< 0.001
TNFα, ng/mg protein	0.0092	0.0056	0.0019	0.001
IL-6, ng/mg protein	0.0026	0.0014	0.0005	< 0.001

### Digestive enzyme activity

In nursery piglets supplemented with CEO, trypsin, lipase, and amylase activities in the duodenum were elevated compared with the Con group (Table 8, *P* < 0.05). Furthermore, dietary CEO supplementation markedly increased the activities of trypsin, lipase and chymotrypsin in the jejunum (Table 8, *P* < 0.05).

**Table 8:** Digestive enzyme activities in intestine of piglets as affected by dietary compound essential oil supplementation

Item	Con	CEO	SEM	*P-value*
Digestive enzyme				
Duodenum				
Trypsin, U/mgprot	46,816	54,215	3,699	0.006
Lipase, U/mgprot	233.27	302.24	34.49	0.003
Amylase, U/mgprot	490.90	533.24	21.17	0.018
Chymotrypsin, U/mgprot	21.52	24.75	1.61	0.104
Jejunum				
Trypsin, U/mgprot	43,393	52,427	4,517	< 0.001
Lipase, U/mgprot	274.23	328.82	27.30	0.008
Amylase, U/mgprot	523.48	533.43	4.98	0.520
Chymotrypsin, U/mgprot	19.81	23.30	1.75	0.042

### Growing Pigs performance

The growth performance parameters for fattening pigs following CEO supplementation during nursery are summarized in Table 9. Growing pigs fed CEOs during nursery showed a numerical increase in BW at 12 and 15 weeks (*P* < 0.05) compared to nursery fed basal diet. At each stage, the ADFI, ADG, and FCR were not significantly different between the two groups.

**Table 9:** Growth performance of subsequent fattening pigs as affected by CEO supplementation in nursery diet^1^

Item	Con^2^	CEO	SEM	*P-value*
Initial BW, kg	29.16	31.47	1.16	0.377
Week 6 BW, kg	64.27	67.20	1.47	0.180
Week 12 BW, kg	98.87	103.30	2.22	0.010
Week 15 BW, kg	119.80	123.9	2.05	0.017
Week 1 to 6				
ADG, g/d	835.85	850.61	7.38	0.999
ADFI, g/d	1654.3	1726.50	36.08	0.939
FCR	0.51	0.49	0.006	0.996
Week 7 to 12				
ADG, g/d	823.81	859.52	17.86	0.997
ADFI, g/d	2300.50	2354.00	26.75	0.979
FCR	0.35	0.36	0.001	0.999
Week 13 to 15				
ADG, g/d	1028.56	1044.44	7.94	0.999
ADFI, g/d	2629.67	2748.00	59.17	0.728
FCR	0.39	0.38	0.005	0.997
Week 1 to 15				
ADG, g/d	863.23	880.24	8.51	0.998
ADFI, g/d	2107.87	2181.80	36.97	0.934
FCR	0.41	0.40	0.003	0.999

^1^ body weight (BW), average daily gain (ADG), average daily feed intake (ADFI), feed conversion ratio (FCR)

^2^ Subsequent control (Con): nursery basal diet. Subsequent CEO: nursery combined essential oil. N = 90 total, n = 45 per group

## Discussion

The nursery period is a key period that connects the lactation period and the growth period, not only to go through the weaning period, but also to lay a good healthy foundation for the need for rapid fattening during the fattening period. Since dietary supplementation has been shown to be a convenient and practical method to effectively avoid oxidative and spoilage reactions induced by microbial growth in the feed, incorporating essential oils into livestock diets is now a common strategy. Moreover, a variety of bioactive compounds contained in essential oils have the potential to serve as multifunctional feed additives for animals, including relieving stress, promoting growth, improving the digestive system environment and resisting pathogen growth [15–17]. However, further research is required to identify their exact role and to determine their routine use in animal production. Consequently, the present study, combined with previous researches, the combination of carvacrol, cinnamaldehyde and thymol was selected to preliminarily explore the effect of compound essential oils on the production performance and intestinal health of piglets in the nursery stage.

Diarrhea during the first 14 days of the nursery period is one of the main factors leading to growth and mortality retardation in nursery pigs [18]. In the current study, no appreciable difference was occurred on diarrhea rate and diarrhea index of piglets in each period of the experiment, but there was a trend of remission. Similarly, Tian et al. [19] revealed that the diarrhea prevalence in piglets fed EOs was lower than that of piglets fed conventional diets. Moreover, this study also revealed that BW on days 14, 42, ADG from days 15 to 28 of the trial and throughout the experimental period were significantly increased in piglets supplemented with CEO, while FCR was also significantly decreased throughout the experimental period. These findings were consistent with those reported by Tian et al. [19] and Z Zeng, X Xu, Q Zhang, P Li, P Zhao, Q Li, J Liu and X Piao [20], who found that EO supplementation increased BW and ADG. The EO may be responsible for the mechanism by inhibiting the proliferation of harmful bacteria in the intestine and reducing intestinal pH. Furthermore, CEO nutritional supplementation boosted growth performance of nursery pigs, particularly in the first stages of the experiment. This finding may be related to the fact that EO lessened the effects of various stressors in the early nursery period via improving intestinal health [21].

Maintaining appropriate intestinal function depends on the integrity of the morphological structure of the intestine. A shortening intestinal villus or an deeper crypt depth decrease the surface area for nutrient absorption, which indicates a reduction of capacity in intestine to absorb nutrients [22]. By assessing VH, CD, and VH:CD ratio, gut morphology can provide some information about gut health [23, 24]. In contrast to the CON group, CEO supplementation in this study enhanced VH, CD, and the VH:CD ratio in nursery pigs. SN Dieguez, JM Decundo, G Martínez, FA Amanto, CP Bianchi, DS Pérez Gaudio and AL Soraci [25] demonstrated that dietary supplemented oregano essential oil improved intestinal architecture. Given that the total luminal villus’ absorptive surface area and the VH:CD ratio have a positive correlation [26], our results suggest suggested that EO treatment can increase the absorption surface area and increase the utilization rate of feed. Besides, Nutrient absorption is also dependent on the activity of digestive enzymes in the gastrointestinal tract, and lowered digestive enzyme activity can impair nutrient digestibility in piglets [27]. Consistent with the study by P Li, X Piao, Y Ru, X Han, L Xue and H Zhang [28], in the current study, dietary supplemented CEO enhanced digestive enzyme activities in duodenum and jejunum.

Nursery piglets are susceptible to issues like slow growth, diarrhea, and impaired intestinal barrier function, which may have a negative impact on their health and growth [29–31]. These factors include weaning stress, dietary changes, environmental changes, and psychological factors. These stressors from the early nursery period also disrupt the structure of the gut microbiota, and increase the proportion of pathogenic bacteria [32, 33]. Intestinal mucosa injury provided a substrate for pathogen propagation, thereby increasing the risk of pathogen adhesion and invasion. Moreover, the toxins and metabolites damaged intestinal mucosal barrier, which resulted in increased intestinal permeability, poor food absorption, and diarrhea in nursery pigs [21]. Therefore, an intact intestinal mucosal barrier for ensuring that provision of adequate dietary nutrition for the whole body and for preventing the invasion of pathogenic bacteria.

Tight junctions are composed of transmembrane barrier proteins (such as claudins and occludins), cytoplasmic scaffold proteins (such as ZO family), and adhesion molecules, were important indicators for evaluating the integrity of the intestinal barrier [34]. These proteins are regarded as essential regulators of paracellular permeability [35]. In the present study, a beneficial effect in duodenum (ZO-1, caudin-1, caudin-5, occludin) jejunum (occluding, E-cadherin) and ileum (ZO-1, caudin-1, caudin-5, E-cadherin) were observed in piglets fed CEO compared to the CON. Pu et al. [36] found that oregano oil administration raised the relative gene expression of claudin-1 and occluding in the jejunum of piglets, which is agreement with our study. It suggested that the improvement of piglet feed utilization and the reduction of diarrhea rate may be related to the improvement of intestinal barrier function. The increasing plasma concentrations of DAO and D-LA are a sign of increased intestinal permeability. DAO, a highly active intracellular enzyme found in the upper villi of the small intestine mucosa, is strongly associated to the production of nucleic acids and proteins by mucosal cells and serves as a marker for the health of the intestinal mechanical barrier and the extent of injury [37, 38]. As a metabolic by-product of bacterial fermentation, D-LA can be produced by many intestinal bacteria. A significant quantity of D-LA generated by intestinal bacteria enters the blood through the injured mucosa when intestinal mucosal permeability rises, raising the serum D-lactic acid level [39]. In the present study, supplementation of CEO to nursery pig diets reduced serum concentrations of DAO, ET and D-LA.

In addition to being a crucial organ for the digestion, absorption, and metabolism of dietary materials, the intestine is also the largest immune organ of the body, housing more than 70% of all immune cells [40]. During the nursery period, the pigs are exposed to a variety of pathogenic and nonpathogenic stimuli, which causes the activation of the gastrointestinal immune system. Numerous pro-inflammatory cytokines are created when the gut immune system is activated, and their excessive production leads to intestinal damage and malfunction. Importantly, gut inflammation is linked to impaired gut growth and development and worse nutrition absorption. Gut chronic inflammatory illnesses are associated with altered gut morphology, reduced ability to absorb nutrients, increased mucosal permeability, damaged mucosa and impaired gut development [41]. Essential oils have been revealed a potential activity for the treatment of inflammatory diseases, especially in chronic inflammatory conditions, with the main mechanism involving reduction of reactive oxygen and proinflammatory cytokines [42]. Animal performance is hampered by inflammatory substances such as IL-1β, TNF, and IL-6 that affect immune and food metabolism [43, 44]. An earlier study demonstrated that a combination of carvacrol and thymol enhanced gut health by lowering TNF-α mRNA expression in the intestine of nursery piglets, and mucosal TNF-α levels are connected with intestinal inflammatory illness [40]. Our results also confirmed that local intestinal inflammation was significantly relieved in nursery pigs after CEO supplementation. Even while this intestinal local inflammation did not cause full-blown clinical symptoms, it significantly reduces growth performance, and essential oils enhanced the health of pigs by regulating the immune response of pigs.

## Conclusions

In conclusion, the inclusion of EOs in diet did not significantly alleviate diarrhea of piglets, but it did improved performance, which could be ascribed to better gut health. 0.1% combination of EOs containing carvacrol, cinnamaldehyde and thymol led to increased gut absorptive area, gut barrier integrity and digestive enzyme activity, alleviative intestine inflammation. In addition, pigs supplemented with essential oil during nursery had better growth performance at the fattening period, thereby verifying the favorable effect of establishing intestinal homeostasis on subsequent growth.

## Methods

The study was carried out in Tengyue Farm, Zhaozhou City, Heilongjiang Province, China. Northeast Agricultural University Institutional Animal Ethics Committee approved the Guide for the Care and Use of Laboratory Animals, which was followed in all animal procedures. The code for ethic approval is NEAUEC20220825.

### CEO product

The product of compound essential oil (CEO) additive was supplied by Weiyuan Animal Pharmaceutical Co., Ltd. (Shijiangzhuang, China). Briefly, whey protein concentrate was dissolved in distilled water to prepare a 5 wt% protein solution; pectin was dissolved in distilled water at 70 ° C under stirring to prepare a 0.5 wt% pectin solution; and maltodextrin was dissolved in distilled water to prepare a 20 wt% maltodextrin solution. The above three solutions were mixed at 1200 rpm for 1 h at a volume ratio of 1:1:1 and stored at room temperature for a period of time to fully hydrate to form a premixed carrier, called whey protein concentrate-pectin complex; the premixed carrier pH was adjusted to 6 with HCl and NaOH. Subsequently, under ultrasonic treatment at 20 kHz, cinnamaldehyde (6.5 kg, > 95% purity), carvacrol (7.2 kg, > 86% purity) and thymol (1 kg, > 95% purity) were added to the premixed carrier (39.2 kg), mixed evenly, and dried at -50 °C for 48 h. The chitosan (50 kg) was heated and melted, sprayed into the fluidized bed by spray gun atomization, coated and granulated with the mixture dried in the previous step, and screened through a 40–60 mesh sieve to obtain CEOs (containing 5% cinnamaldehyde, 5% carvacrol, and 0.8% thymol).

### Animals, housing, and diets

The study was divided into two digestibility trials. In trial 1, 100 cross-bred (Landrace × Large White) piglets weaned at 28-d old (8.08 ± 0.34 kg of body weight) were housed in ten pens (3 m × 2 m × 0.7 m) with a leaky sprayed plastic floor with a rectangular plastic adjustable slot and a duckbill waterer for 10 piglets per pen. The following two experimental diets were given to the piglets: a control diet (Con) fed with basal ration only; a diet with a 0.1% replacement additive consisting of CEO. The CEO dose was selected based on previous studies [13, 45]. In the 42d trial, 100 piglets were randomly allotted to the 2 previously described dietary treatments according to body weight and gender, each consisting of 50 piglets in 5 replicate pens per group and 10 piglets per pen. The levels of the nutrition met or exceeded the NRC (2012) standard, and the ingredient of the basic diet is shown in Supplementary Table S1. The feed was fed in three phases on average for 6 weeks. The piglets are periodically dewormed and immunized. Overall, the piglets were given powder and permitted to feed and drink as they pleased. Mortality of piglets was zero during the experiment.

To investigate the growth performance of the nursing pigs supplemented with CEO during the fattening period. In trial 2, a total of 45 growing pigs from each group remaining after slaughter were used in the subsequent experiments, with an average weight of 29.16 ± 0.27 kg in the control group and 30.81 ± 0.64 kg in the CEO group. There were 9 replication pens, with 5 pigs per pen. All growing pigs were fed the same diet. The diets were fed in 3 phases, including a growing phase from weeks 1 to 6, an early finishing phase from weeks 7 to 12, and a late finishing phase from weeks 13 to 15. The diets were formulated to meet or exceed NRC (2012) nutritional requirements (Supplementary Table S2).

### Growth performance determination

Piglets and feeders were weighted on d 0, 14 and 28. The body weight (BW) of the pigs were calculated for each feeding phase and for the whole experimental period. In addition, discarded feed from each pen was collected daily from the floor near the feeder, weighed, and subtracted from given for calculation of average daily feed intake (ADFI). And the average daily gain (ADG), ADFI and feed conversion ratio (FCR; FCR = ADFI/ADG) were calculated on a pen basis. The diarrhea rate was calculated as follows: Diarrhea rate (%) = (number of diarrhea weaned pigs)/(total number of experimental weaned pigs × experimental time (d)) × 100%. Diarrhea index = total fecal scores/total number of experimental weaned pigs.

To evaluate the index and rate of diarrhea, fecal consistency was assessed visually twice daily throughout the experimental period by a fixed observer blinded to treatment. The severity of diarrhea was evaluated using the following scoring system: 1 = normal feces; 2 = possible slight diarrhea; 3 = fluid feces; 4 = very watery diarrhea. Feces were observed as shown in Supplementary Fig. S1. The occurrence of diarrhea was defined as maintaining fecal scores of 3 or 4 for 2 consecutive days.

In the observation of fattening period, growing pigs and feeders were weighted at week 6, 12 and 25. The BW of the pigs were recorded, and ADG, ADFI and FCR were calculated on a pen basis during the growing phase, early finishing phase, late finishing phase, and the whole finishing period.

### Experimental procedures and sample collection

On d 42, one piglet from each pen (n = 5) was randomly selected and euthanized for the collection of serum, small intestine tissues and content, colon mucosa. Pigs were given a 12-hour fast before being euthanized by electric shock and exsanguination, and tissue and serum samples were collected immediately. The organ coefficients of the liver, spleen, and kidney, as well as the relative length of the small intestine, were calculated after the liver, spleen, and lung were weighed on an electronic scale and measured in length. For histological investigation and scanning electron microscopy (SEM), the intestine segments were discretely collected, washed with ice-cold physiological saline or PBS, and then preserved in 4% paraformaldehyde or 2.5% glutaraldehyde. Meanwhile, the pH levels of the contents of the mid-duodenum, mid-jejunum, mid-ileum, mid-caecum, and mid-colon were ascertained using a portable pH detector (testo 205, Chunan Electronic Co., Ltd., Shanghai, China). The contents of the jejunum and ileum from other segments of the intestine were collected and stored at -80 °C. The serum was subtly extracted and stored at -80 °C after blood samples from the vena jugularis were collected into vacuum blood tubes and centrifuged at 3,000 × g for 30 min.

### Histological and morphometric analysis

The paraffin-embedded tissue sections were stained with hematoxylin and eosin (H&E), according to the methods of our previous study [46]. The crypt depth (CD) is calculated as the distance from the bottom of the crypt to the crypt-villus junction. The villus height (VH) is measured as the distance from the crypt-villus junction to the tip of the villus. For each group, 8 villi from each sample were measured at least.

### Scanning electron microscope (SEM)

The tissues were immersed in 2.5% glutaraldehyde for 24 h at 4 °C, then rinsed in PBS and treated for 1 h in sodium cacodylate buffer with 1% osmium tetroxide. Next, the samples are sequentially dried to critical point in alcohol solvent and liquid CO2 under pressure. Subsequently, the samples were pasted to stubs by carbon tape and covered with gold. The images were captured by scanning electron microscope (EVO MA 15, Carl Zeiss AG, Jena, Germany).

### Biochemical parameter analysis

To detect local intestinal inflammation, 0.1 g of colonic mucosal homogenate was diluted with PBS (1 mL). Subsequently, the interleukin-1 beta (IL-1β), tumor necrosis factor alpha (TNF-α) and interleukin-6 (IL-6) levels in colonic mucosal samples were quantified by enzyme-linked immunosorbent assay (ELISA) kits (IL-1β: Cat No. AD0137Po; TNF-α: Cat No. AD0073Po; IL-6. Cat No. AD0132Po; Beijing Chenglin Biotechnology Co., Ltd., Beijing, China) with microplate reader (ELx808, BioTek Instruments, Inc., Vermont, USA). Trypsin (Ultraviolet colorimetry), lipase (Microplate method) and amylase (Starch-iodine colorimetry) from intestinal content were measured by the commercial ELISA kits (Nanjing Jiancheng Bioengineering Institute, Nanjing, China). And according to the producer’s explanation, chymotrypsin from intestinal content was measured using ELISA kits (Beijing Chenglin Biotechnology Co., Ltd., Beijing, China). Under the producer’s expound, serum diamine oxidase (DAO), D-lactic acid (D-LA) and endothelin (ET) were quantified using an available enzyme-linked immunosorbent assay (ELISA) kit (Beijing Chenglin Biotechnology Co., Ltd., Beijing, China).

### Real time-quantitative PCR

Real-time quantitative PCR was used to assess the expression of the tight junction protein-related genes ZO-1, Claudin-1, Claudin-5, Occludin and E-cadherin in the duodenum, jejunum, and ileum. The Supplementary Table S3 lists the primers utilized in this study. Total RNA extracted with TRIzol (Beyotime Institute of Biotechnology, Shanghai, China) was reverse transcribed into cDNA using a reverse transcription kit (TaKaRa Biotechnology Co., Ltd., Tokyo, Japan). The target gene transcript levels were normalized to β-actin. qPCR was performed in a 10 µL reaction volume with a sequence detection system (Applied Biosystems, Foster City, CA) using SYBR Green (TaKaRa Biotechnology Co., Ltd.). The data were analyzed following the 2^−ΔΔCt^ method.

### Statistical analysis

Excel 2019 was used for raw data statistics, SPSS 19.0 (version 19.0, SPSS Inc., Chicago, IL, USA) was used for one-way ANOVA analysis between groups. The replicate (pen) was considered as a unit to analyze the data concerning growth performance and apparent digestibility of nutrients in piglets, the diarrhea rate of piglets was analyzed by the chi-square test. Individual piglet was considered as a unit to analyze the data concerning serum, digestive enzymes, intestinal morphology and so on. The results are expressed as the mean and standard error of mean (SEM). Level of significance was set at P < 0.05, whereas level of 0.05 < P < 0.1 was considered to have such a tendency.

## Electronic supplementary material

Below is the link to the electronic supplementary material.

Supplementary Material 1

## Data Availability

Data related to this study can be accessible by request to the corresponding author.
