# Supplementary material for: Effects of dietary supplementation with a carvacrol–cinnamaldehyde–thymol blend on growth performance and intestinal health of nursery pigs
Source: Porcine Health Manag. 2023 May 23;9:24. doi: 10.1186/s40813-023-00317-x (PMC10207703; doi:10.1186/s40813-023-00317-x)
Supplement: Supplementary file 1 — Supplementary Material 1 [file 40813_2023_317_MOESM1_ESM.docx]

**Effects of dietary supplementation with a carvacrol - cinnamaldehyde - thymol blend on growth performance and intestinal health of nursery pigs**

Bi-Chen Zhao^1^, Tian-Hao Wang^1^, Jian Chen^1^, Bai-Hao Qiu^1^, Ya-Ru Xu^1^, Qing Zhang^4^, Jian-Jie Li^4^, Chun-Jiang Wang^4^, Qiu-Feng Nie^4^, Jin-Long Li^1,^2^,3,^*

^1^*College of Veterinary Medicine, Northeast Agricultural University, Harbin, 150030, P. R. China.*

^2^*Heilongjiang Key Laboratory for Laboratory Animals and Comparative Medicine, Northeast Agricultural University, Harbin, 150030, P. R. China.*

^3^*Key Laboratory of the Provincial Education Department of Heilongjiang for Common Animal Disease Prevention and Treatment, Northeast Agricultural University, Harbin, 150030, P. R. China.*

^4^ Weiyuan Animal Pharmaceutical Co., Ltd. Shijiangzhuang, *052165, P. R. China.*

*Corresponding author:

E-mail address: Jinlongli@neau.edu.cn (J.L. Li)

**Supplementary Table S1.**

Ingredient composition of experimental diets for nursery pigs, as-fed^1^

| Ingredient, % as- fed | Phase 1 | Phase 2 | Phase 3 |
| --- | --- | --- | --- |
| Corn, CP 7.6% | 53.80 | 57.10 | 66.00 |
| Soybean meal, 46.5% CP | 24.70 | 27.90 | 30.00 |
| Whey powder, CP 3.8% | 10.00 | 10.00 | 10.00 |
| Fermented soybean meal, CP 51% | 4.00 | 4.00 | 4.00 |
| Angel yeast, CP 51.3% | 3.00 | 2.00 | - |
| Fish meal, CP 65.3% | 4.00 | - | - |
| Soybean oil | 1.50 | 0.64 | 0.16 |
| Salt | 0.65 | 0.62 | 0.59 |
| Dicalcium phosphate | 0.79 | 0.65 | 0.62 |
| Limestone | 0.92 | 1.19 | 1.16 |
| L-Lysine HCl, 78% | 0.51 | 0.55 | 0.41 |
| DL-Methionine, 98% | 0.10 | 0.09 | 0.08 |
| Threonine, 98% | 0.16 | 0.15 | 0.14 |
| L-Tryptophan, 98% | 0.07 | 0.06 | 0.05 |
| Trace mineral premix^2^ | 0.15 | 0.15 | 0.15 |
| Vitamin premix^3^ | 0.05 | 0.05 | 0.05 |
| Analyzed composition |  |  |  |
| Dry matter, % | 89.9 | 90.2 | 90.8 |
| Crude fiber, % | 2.60 | 2.75 | 3.00 |
| Ash, % | 5.74 | 5.81 | 5.63 |
| Net energy, Mcal/kg | 2.498 | 2.461 | 2.430 |
| Acid detergent fiber, % | 3.05 | 3.10 | 3.10 |
| Neutral detergent fiber, % | 6.52 | 6.41 | 7.80 |
| Crude protein, % | 21.61 | 20.21 | 19.70 |

^1^The experimental diets were fed in 3 phases; Phase 1 from d 0 to 14, Phase 2 from d 14 to 28, and Phase 3 from d 28 to 42.

^2^Provided the following per kilogram of diet: 11,011 IU vitamin A, 1,652 IU vitamin D3, 55 IU vitamin E, 0.04 mg vitamin B12, 4.4 mg menadione, 9.9 mg riboflavin, 61 mg pantothenic acid, 55 mg niacin, 1.1 mg folic acid, 3.3 mg pyridoxine, 3.3 mg thiamine, and 0.2 mg biotin..

^3^ Provided the following per kilogram of diet: 165 mg Zn as ZnSO4, 23 mg Fe as FeSO4; 17 mg Cu as CuSO4, and 44 mg Mn as MnSO4.

**Supplementary Table S2.**

Ingredient composition of experimental diets growing and finishing pigs, as-fed^1^

| Ingredient, % as- fed | Growing  (1-6 weeks) | Early finishing  (7-12 weeks) | Late finishing  (13-15weeks) |
| --- | --- | --- | --- |
| Corn, CP 7.6% | 59.05 | 63.76 | 69.02 |
| Soybean meal, 46.5% CP | 35.39 | 9.95 | 5.50 |
| Wheat bran | 10.00 | 10.00 | 10.00 |
| Wheat | 10.00 | 10.00 | 10.00 |
| Soy hulls | 1.00 | 1.26 | 1.87 |
| Soybean oil | 2.57 | 2.00 | 1.38 |
| CaCO3 | 0.82 | 0.73 | 0.53 |
| Monocalcium phosphate | 0.93 | 0.78 | 0.54 |
| L-Lysine HCl, 78% | 0.57 | 0.52 | 0.33 |
| DL-Methionine, 98% | 0.11 | 0.07 | - |
| Threonine, 98% | 0.16 | 0.14 | 0.07 |
| L-Tryptophan, 98% | 0.06 | 0.05 | 0.03 |
| Vitamin premix^1^ | 0.05 | 0.50 | 0.50 |
| Solt | 0.23 | 0.23 | 0.24 |
| Analyzed composition |  |  |  |
| Dry matter, % | 86.5 | 86.9 | 86.1 |
| Net energy, Mcal/kg | 4.283 | 4.266 | 4.263 |
| Crude protein, % | 14.56 | 13.04 | 11.23 |
| Crude fat, % | 5.50 | 5.05 | 4.55 |
| Crude fiber, % | 3.62 | 3.57 | 3.63 |
| Ash, % | 4.33 | 3.94 | 3.36 |
| Neutral detergent fiber, % | 8.9 | 10.1 | 10.6 |
| P, % | 0.58 | 0.53 | 0.47 |
| Ca, % | 0.66 | 0.59 | 0.46 |

^1^Provided the following per kilogram of diet: retinyl acetate, 10,000 IU; cholecalciferol 2500 IU; dl-tocopherol acetate, 50 IU; menadione, 5.0 mg; thiamin, 2.0 mg; riboflavin, 5.0 mg; pantothenic acid, 12.0 mg; pyridoxine, 10.0 mg; niacin, 30.0 mg; biotin, 0.2 mg; folic acid, 1.5 mg; cyanocobalamin 0.05 mg; choline chloride 1500 mg; iron, 100 mg; copper, 20 mg; manganese, 25 mg; zinc, 100 mg; selenium, 0.3mg; iodine, 0.3mg.

**Supplementary Table S3**

Sequences of oligonucleotide primers for qRT-PCR.

| Gene Names | Sequence(5' → 3') | NCBI Reference Sequence | Amplicon size (bp) |
| --- | --- | --- | --- |
| β-actin | GACGATATTGCTGCGCTCGT  TAGGAGTCCTTCTGGCCCAT | XM_021086047.1 | 152 |
| ZO-1 | ACTTGTCAGCTCAGCCAGTC  ACAGGCCTCAGAAATCCAGC | XM_021098896.1 | 82 |
| Claudin-1 | CGTAGCATCCTGGAGCAGTC  AGTCTGTGCCAATTGAGGCT | XM_005670262.3 | 122 |
| Claudin-5 | CGAGTTCTACGACCCGACTG  GGGCCGAATACTTGACAGGG | NM_001161636.1 | 173 |
| Occludin | CTCGTCCAACGGGAAAGTGA  ACGCCTCCAAGTTACCACTG | NM_001163647.2 | 155 |
| E-cadherin | GTGGTTCCGAAGCTGCTAGT  CCCCACTCGTTCAGGTAGTC | NM_001163060.1 | 85 |


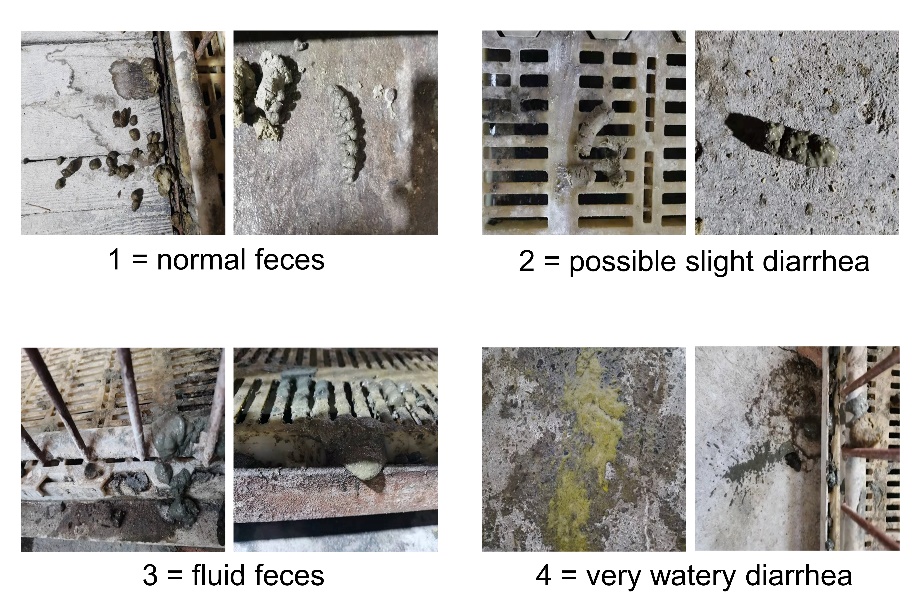


**Supplementary Fig. S1.** Representative fecal images.


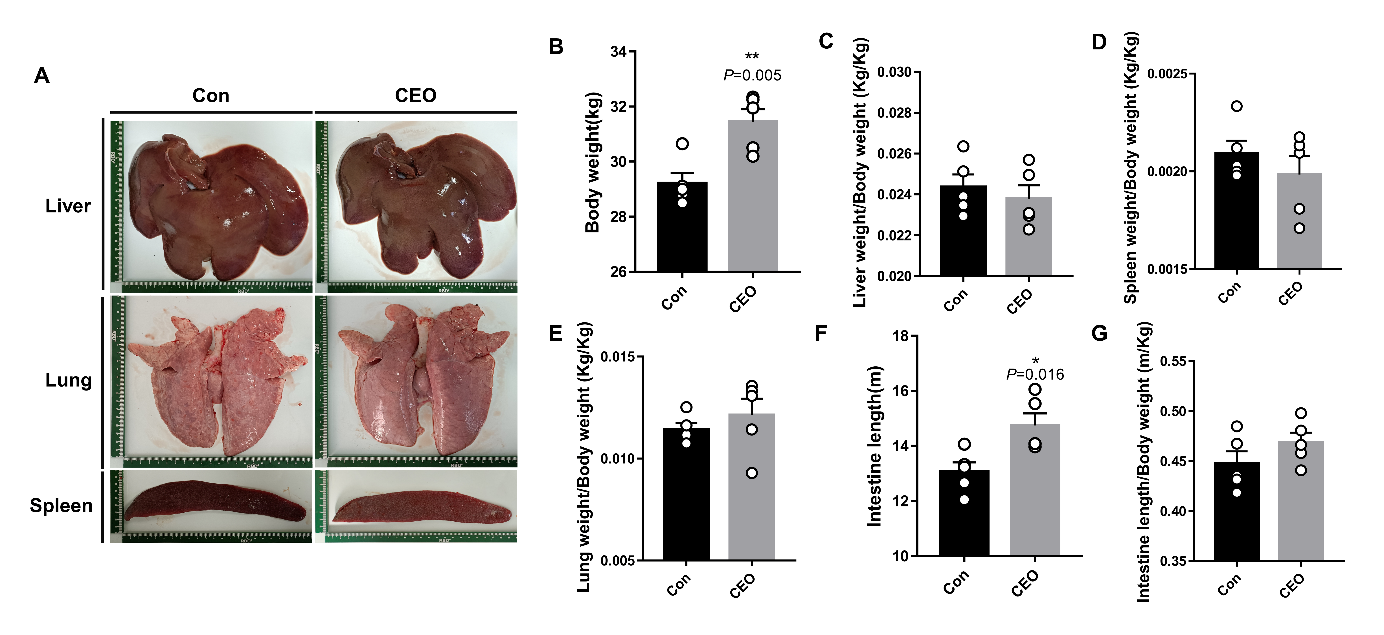


**Supplementary Fig. S2.** Effects of plant essential oils on the organs of nursery pigs. (A) Organ figure of liver, lungs and spleen; (B) Body weight; (C) Organ coefficient of the liver; (D) Organ coefficient of the spleen; (E) Organ coefficient of the lung; (F) Intestinal length; (G) Intestinal length / Body weight. Data were presented as means ± SEM. *P<0.05, **P<0.01.
